# Preferences between three options for androgen deprivation therapy: a focus group study

**DOI:** 10.1111/bju.16863

**Published:** 2025-07-30

**Authors:** Hannah L. Rush, Sophie Merrick, John Marshall, John Deighan, Hoda Abdel‐Aty, Abdulla Alhasso, Noel W. Clarke, Roger Kockelbergh, Stephen Mangar, Stuart D. Rosen, Nicholas D. James, Duncan C. Gilbert, Annabelle South, Ruth E. Langley

**Affiliations:** ^1^ MRC Clinical Trials Unit at UCL, Institute of Clinical Trials and Methodology University College London London UK; ^2^ Guy's and St Thomas’ Foundation Trust London UK; ^3^ The Institute of Cancer Research London UK; ^4^ The Royal Marsden NHS Foundation Trust London UK; ^5^ National Heart and Lung Institute Imperial College London London UK; ^6^ Imperial College London London UK; ^7^ Patient and Public Representatives for the PATCH Trial London UK; ^8^ The Beatson West of Scotland Cancer Centre Glasgow UK; ^9^ University of Manchester Manchester UK; ^10^ The Christie Hospital NHS Foundation Trust Manchester UK; ^11^ Salford Royal Hospital Manchester UK; ^12^ University Hospital of Leicester NHS Trust Leicester UK

**Keywords:** androgen deprivation therapy, transdermal oestradiol, relugolix, LHRH agonists, patient choice, treatment decision‐making, focus group study

## Abstract

**Objectives:**

Androgen deprivation therapy (ADT) forms the mainstay of treatment for advanced prostate cancer. Traditionally administered as a luteinising hormone‐releasing hormone (LHRH) agonist depot injection, newer options for ADT include transdermal oestradiol patches (tE2) or oral LHRH antagonists. This study aimed to identify whether this is an important choice for men, which treatment men would choose if offered either LHRH agonist injections, tE2 patches or oral LHRH antagonists as ADT, and to explore the factors influencing this decision.

**Subjects, Patients, and Methods:**

Five focus groups were conducted. A total of 24 men from around the UK participated in discussions, of whom 10 had never had prostate cancer and 14 had early prostate cancer but had not received ADT. Focus groups were co‐facilitated with patient and public involvement representatives. Transcripts were analysed using a critical realist thematic analysis approach.

**Results:**

Participants reported that having a choice of ADT is important and being involved in making treatment decisions can help men maintain an element of control. Most men expressed a preference to avoid ADT with LHRH agonist injections; 14 of the 24 men reported they would choose an oral LHRH antagonist, eight reported they would choose tE2 patches, and two that they would choose LHRH agonist injections. Participants reported a large number of factors that influenced their treatment choice that were grouped into: (i) side‐effects of treatment, (ii) logistical aspects, and (iii) advice from trusted others. Personal preferences between different types of ADT were based on factors that participants identified as important to them, this prioritisation of factors was influenced by participants’ past experiences, current health beliefs, and future expectations.

**Conclusions:**

Men wish to have choice of ADT, and many would not choose LHRH agonists injections if there were other ADT options available. This should be considered, as reimbursement committees and guideline‐makers consider the role of alternative ADT strategies.

**Abbreviations**
ADT: androgen deprivation therapy
FGD: focus group discussion
PATCH: Prostate Adenocarcinoma TransCutaneous Hormone (trial)
PPI: patient and public involvement
STAMPEDE: Systemic Therapy in Advancing or Metastatic Prostate Cancer: Evaluation of Drug Efficacy (trial)
tE2: transdermal oestradiol

## Introduction

An estimated 1.4 million men worldwide were newly diagnosed with prostate cancer in 2020 [1]. Many men are diagnosed with advanced prostate cancer, [2] where androgen deprivation therapy (ADT) provides the backbone of treatment regimens. LHRH agonists, administered as a depot injection, are the most common form of ADT used in high‐income countries and effectively suppress testosterone in >90% of men. Newer options for use as ADT include the oral LHRH antagonist (relugolix), and transdermal oestradiol (tE2) patches, re‐purposing hormone replacement therapy patches recommended for women experiencing menopausal symptoms.

Men with prostate cancer treated with either LHRH agonist injections, oral LHRH antagonist, or tE2 patches have similar rates of castration [3, 4], a surrogate marker of efficacy of ADT [5]. Recently, the Phase III Prostate Adenocarcinoma TransCutaneous Hormone (PATCH; ClinicalTrials.gov identifier NCT00303784) and Systemic Therapy in Advancing or Metastatic Prostate Cancer: Evaluation of Drug Efficacy (STAMPEDE; NCT00268476) trials demonstrated that in addition to similar castration rates, men treated with tE2 patches have non‐inferior prostate cancer outcomes in comparison to men treated with subcutaneous LHRH analogues [6].

LHRH agonists and antagonists, whether administered parenterally or orally, have similar mechanisms of action lowering both testosterone and oestradiol, and the side‐effects of these two approaches are generally considered comparable. A potentially important difference was noted in the Phase III HERO trial (NCT03085095) comparing relugolix (currently the only licensed oral LHRH antagonist) with subcutaneous injected LHRH agonists, where a lower rate of major adverse cardiovascular events was observed in patients treated with relugolix [3]. Ongoing trials aim to confirm this observation and quantify the magnitude of any cardiovascular benefits [7, 8].

Conversely, tE2 reduces testosterone production by increasing oestradiol levels. As suppression of oestradiol contributes to many of the side‐effects, including loss of bone mineral density, adverse metabolic profiles, and hot flushes associated with LHRH analogues, tE2 abrogates these oestradiol related side‐effects. Importantly, transdermal administration of oestradiol avoids the excess cardiovascular risk associated with oral oestrogens [4].

Consequently, it is likely that patients and clinicians will make choices between treatment‐based aspects such as adverse events, impact on quality of life, route of drug administration, and persistence of castration after completing treatment. There is currently no research to indicate whether men would value having a choice between these different types of ADT, and if so whether any of the treatment options would be preferrable to men.

This qualitative focus group study presented men with information about each of the three treatments (LHRH agonist injections, oral LHRH antagonist, or tE2 patches) within focus group discussions (FGDs) and then asked them to report which of the three types of ADT they would choose. Their ADT choice was used as a springboard to further explore the range and breadth of opinions around aspects of this treatment decision, including the importance of choice in this setting and the factors participants reported influenced their decision‐making.

## Subjects, Patients, and Methods

### Research Team

The research team consisted of three oncology doctors (H.L.R., S.M., and H.A.) and one non‐clinical doctor (A.S.). Two patient and public involvement (PPI) representatives (J.M. and J.D.) with lived experience of prostate cancer were involved throughout the study.

### Participants

This study explored the opinions of men aged >50 years (chosen to be reflective of men at risk of developing prostate cancer). This included two discrete populations, one group who previously or currently had prostate cancer, yet had not received ADT, and a second group who had never been diagnosed with prostate cancer.

### Design

Men were recruited to participate in audio‐visual FGDs conducted on‐line via Microsoft Teams(Microsoft, Redmond, WA, USA) between January to November 2023. Recruitment was predominantly via on‐line advertisements placed in prostate cancer‐specific websites and on the National Institute for Health and Care Research (NIHR) People in Research website, but snowball recruitment (where participants were invited to share the details of the FGD with appropriate associates) and printed posters were also used. A £25 voucher was offered to reimburse participants for their time [9]. Potential participants completed an electronic consent form and baseline characteristics questionnaire.

Five focus groups were held sequentially, allowing time for analysis between discussions, until there was adequate information to address the research question [10]. Therefore, sample size was not defined strictly a priori as it was dependent on the data received. Sequential analysis meant subsequent sessions could be adapted to explore aspects that emerged during previous talks in more detail. Each group was planned with a maximum of six men to allow sufficient time for contribution from every participant and discussion was limited to 90 min. Each FGD was co‐facilitated by two oncologists (H.L.R. plus S.M. or H.A.) and a PPI representative (J.M. or J.D.). FGDs were held separately with men who had been diagnosed with prostate cancer and those who had not.

A topic guide was used to facilitate each FGD (Appendix S1). During each discussion participants were shown a 15‐min presentation with analogous information about each of the three types of ADT, including effectiveness, how treatment is administered including supportive medications and additional tests, and common and occasional side‐effects (Appendix S2). This was designed to reflect the type of information given to a patient starting a new treatment.

After the initial presentation, discussion was led by the PPI representatives. Participants were asked if they were in a hypothetical situation where they required ADT, whether they had a preference for a particular ADT and the reasons for their choice.

Each FGD was recorded on Microsoft Teams and automated transcripts of the discussion were generated. Automated transcripts were anonymised by H.L.R. and reviewed to ensure they were accurate. A naturalised transcription style was implemented in which conversation fillers (e.g., ‘umm’ and ‘err’) and perseverations were removed from the transcript [11]. Anonymised transcripts were coded in the NVivo version 2020 software package (NVivo qualitative software, Lumivero, Melbourne, Victoria, Australia) and by hand with paper copies. Transcripts were not sent for review by participants; however, the themes developed following analysis were discussed with the PPI representatives who had each co‐facilitated at least two FGDs.

### Analysis

A critical realist approach to thematic analysis was used to explore whether casual reasons for men's choices could be identified from the data [12]. After familiarisation with the transcript, sections of text were labelled with codes by H.L.R., which were standardised and consolidated where possible. In addition, excerpts of the transcripts were reviewed and coded by A.S. and S.M. in data clinics. Codes were explored for structure and hierarchy and then reviewed to ensure they maintained descriptive and interpretative validity. During the coding process, themes were developed, which looked to address the original research questions. Themes were also reviewed for validity. Coding and themes were developed iteratively until it was felt that plausible and reasonable explanations to the research questions had been reached.

### Ethics

The study received ethical approval from University College London Research Ethics Committee on the 16 November 2022 (23 797/001). The study was conducted and reported according to the Standards for Reporting Qualitative Research checklist [13] (Appendix S3). Data were managed according to the principles of General Data Protection Regulation.

## Results

A total of 24 men were included in five FGDs, 14 of whom had been diagnosed with prostate cancer and 10 who had never been diagnosed with prostate cancer. Men from around the UK (Scotland, Wales, and England) participated, including participants from rural, suburban, and urban areas. Of the cohort, 20 self‐identified as White, three identified as Asian, and one as White and Caribbean.

Key baseline characteristics are presented according to participant's choice of ADT in Table 1. During discussions, treatments were referred to as ‘injections’ (LHRH agonist injections), ‘tablets’ (LHRH antagonist tablets) and ‘patches’ (tE2 patches), the same terminology is used below.

**Table 1:** Background characteristics presented per primary choice of ADT treatment.

Variable, *n/N* (%)	First choice of ADT			Total
	Injections (*n* = 2)	Tablets (*n* = 14)	Patches (*n* = 8)	All (*N* = 24)
**Prostate cancer**				
Yes	0/2	9/14	5/8	14/24 (58)
No	2/2	5/14	3/8	10/24 (42)
**Age, years**				
50–59	1/2	5/14	2/8	8/24 (33)
60–69	1/2	4/14	0/8	5/24 (21)
70–79	0/2	5/14	5/8	10/24 (42)
≥80	0/2	0/14	0/8	0/24 (0)
Missing	0/2	0/14	1/8	1/24 (4)
**Ethnicity**				
White	2/2	11/14	6/8	19/24 (79)
Asian	0/2	2/14	1/8	3/24 (13)
White/Caribbean	0/2	1/14	0/8	1/24 (4)
Missing	0/2	0/14	1/8	1/24 (4)
**Living environment**				
Rural	2/2	1/14	2/8	5/24 (21)
Suburban	0/2	4/14	3/8	7/24 (29)
Urban	0/2	9/14	2/8	11/24 (46)
Missing	0/2	0/14	1/8	1/24 (4)
**Employment**				
Retired	2/2	8/14	5/8	15/24 (63)
Working	0/2	3/14	1/8	4/24 (17)
Carer	0/2	1/14	0/8	1/24 (4)
Other	0/2	1/14	1/8	2/24 (8)
Missing	0/2	1/14	1/8	2/24 (8)
**Other significant co‐morbidities**				
Yes	1/2	4/14	2/8	7/24 (29)
No	1/2	10/14	5/8	16/24 (67)
Missing	0/2	0/14	1/8	1/24 (4)
**Time to travel to GP surgery, min**				
<15	1/2	11/14	4/8	16/24 (67)
≥15	1/2	3/14	3/8	7/24 (29)
Missing	0/2	0/14	1/8	1/24 (4)
**Time to travel to nearest cancer centre, min**				
<30	1/2	8/14	2/8	11/24 (46)
≥30 min	1/2	6/14	3/8	10/24 (42)
Missing	0/2	0/14	3/8	3/24 (13)

Five themes (with representative quotations listed in Table 2), were identified during analysis of the FGDs. Participants are labelled as either ‘P’ (from the group who have or previously had prostate cancer) or ‘NP’ (from the group of men who never had prostate cancer).

**Table 2:** Themes, codes, and illustrative quotations.

Theme	Codes	Illustrative quotation
1: Having a choice of ADT options is important for men	Choice is important Feeling of control Clinician‐led decision Shared decision‐making	‘Choice gives you more agency. I suppose the feeling you got more agency, however little control over your disease, I mean, must be significant’. NP8 ‘I think we as patients need to have more of that choice’. P5 ‘I would go with what the doctor says. They’re the experts’. P6 ‘I think it should be a shared decision not the clinician's decision. I see the role of the clinician as leading the discussion and sharing the information and discussing the pros and cons, but ultimately it should be a joint decision between what your preference is and what your thoughts are based on the information that you've been given’. NP9
2: Participants perform an individual risk–benefit analysis to guide their treatment decisions, with most men choosing to avoid standard hormone injections	Decisions depends on situation	‘If it was going to be a short course and curative, you would accept a more radical set of complications or potential side effects’. P8 See Table 3 for more illustrative quotes for Theme 2
3: Anticipated adverse effects of treatment (including side‐effects, treatment delivery and psychological impacts) influence which option is preferred	Injections are stronger Fear of side‐effects Physical side‐effect – heart Physical side‐effect – metabolic syndrome Physical side‐effect – hot flushes Physical side‐effect – gynaecomastia Treatment reversibility Psychological side‐effects	‘Everything I’ve had that which has been an injection has been more intense than something which is taken by tablet form’. NP3 ‘The fact that the tablet has got a less of an impact on heart problems makes that attractive’. P8 ‘My blood pressure is already raised … I’m gonna go for the patches because of the metabolic syndrome and that should reduce the risk’. NP8 ‘What I like about perhaps the tablet and the patches is they’re easily reversible … I like the idea of being able to sort of step out of a treatment if you know if the side effects are unbearable’. P4 ‘Hot flushes being less is actually quite important cause they can be a real pain and that's why I leaned toward the patches rather than the tablets’. NP9 ‘[I] don’t like the idea of more breast tissue’. NP10 ‘I wouldn’t really want a patch reminding me every moment of the day’. NP8 (Talking about his partner) ‘Opening that silver foil every morning and night and taking that tablet reminds her every day that she has the BRCA gene…and that's why I wouldn’t take a tablet’. NP6 ‘I really hate injections’. P10
4: Logistical issues (control, convenience, confidence, and cost to the health system) influence which option is preferred	Control – treatment administration Convenience Confidence Cost	‘But the patches in many respects, it's something that you’re physically doing yourself … you’re actually I suppose going through some form of treatment that's tangible’. P10 ‘I would just prefer to have some control’. P15 ‘I’d certainly discount the injections for me. Being a busy boy, going to GP or hospitals periodically is time consuming and difficult at times, so I kind of discount that one’. P7 ‘I’d go for tablets first as they are convenient and they are easy’. P12 ‘This business of two, three, or four patches changing them two or three times a week sounds not as consistent as taking one tablet a day’. P6 ‘I didn’t realise it (cost) was that massively different. Might have affected my choice, if I had been given that at the beginning’. P8
5: Advice from trusted others supports men making treatment decisions	Partner supports decisions Independent decisions Charities support men	‘My wife would be involved in any decision making, because she's the one that's going to be there looking after me if anything goes wrong’. NP7 ‘My wife came with me to all the meetings, and we talked. But you know the decisions were left to me’. P8 (PCUK) ‘… have a service that others may have used where you can phone up and speak to a nurse. And that was hugely helpful. I phoned up a couple of times and got some more information’. P9 ‘I looked a lot on the website and took a bit out of Macmillan’. P6

NP(participant number), from the group of men who never had prostate cancer; P(participant number), from the group who have or previously had prostate cancer; PCUK, Prostate Cancer UK.

### Theme 1: having a choice of ADT options is important for men, but there is variability in how much men wish to lead the treatment decision

The majority of men reported that they felt having a choice of ADT would be important. In this discussion, based on the explicit premise that all treatments were equally efficacious, having a choice between these three types of ADT offers alternatives for modes of treatment delivery and the risk of developing certain side‐effects. An alternate opinion from one participant without prostate cancer was that there was insufficient difference between the three treatments to justify the necessity of a choice being available. Men reported that being involved with treatment decisions would give them a feeling of control over their healthcare.

**Fig. 1:**
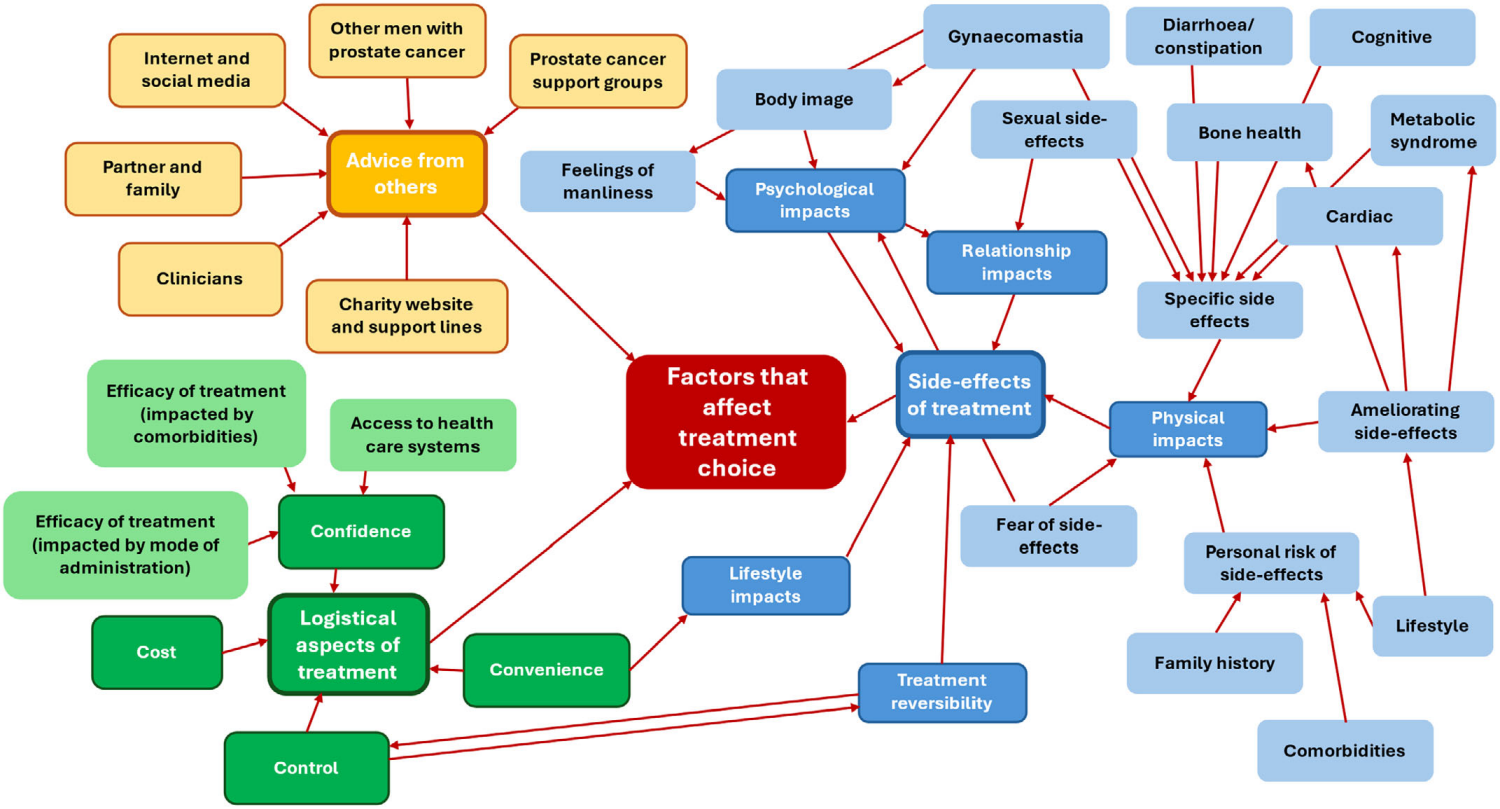
A diagram of factors influencing treatment choice in this study. Three main categories were identified: side‐effects of treatment (blue), advice from trusted others (yellow), and logistical factors (green). Lighter colours represents lower level codes, whilst darker colours reflect code clusters or themes.

The degree of agency men wished to take in leading the treatment decision was variable. Some men reported they would expect a clinician to make a treatment recommendation for them, considering all aspects of a man's health. Others reported they would expect clinicians to present them with a summary of the evidence and allow them to make the decision. A challenge with this approach is that participants valued different aspects of the treatments, and a wide range of factors was cited influencing treatment choices (Fig. 1).

### Theme 2: participants perform an individual risk–benefit analysis to guide their treatment decisions, with most men choosing to avoid standard hormone injections

During discussions men were divided between which type of ADT they would choose. Men preferred to avoid standard hormone injections; 14 men reported they would choose tablets, eight men reported they would choose patches, with only two men reporting they would choose injections as their first choice for ADT. Men felt that a decision between these types of ADT might depend on the situation, e.g., dependent on ADT duration or treatment intent (neoadjuvant/adjuvant vs palliative). The factors that were cited as influencing treatment choices could be broadly grouped into three categories; each discussed in more detail below but summarised as side‐effects, logistics, and advice from others. A comparison of the demographic features of groups of men who made different ADT choices did not identify any pattern that could be used to predict which treatment an individual would pick, although groups were small.

**Fig. 2:**
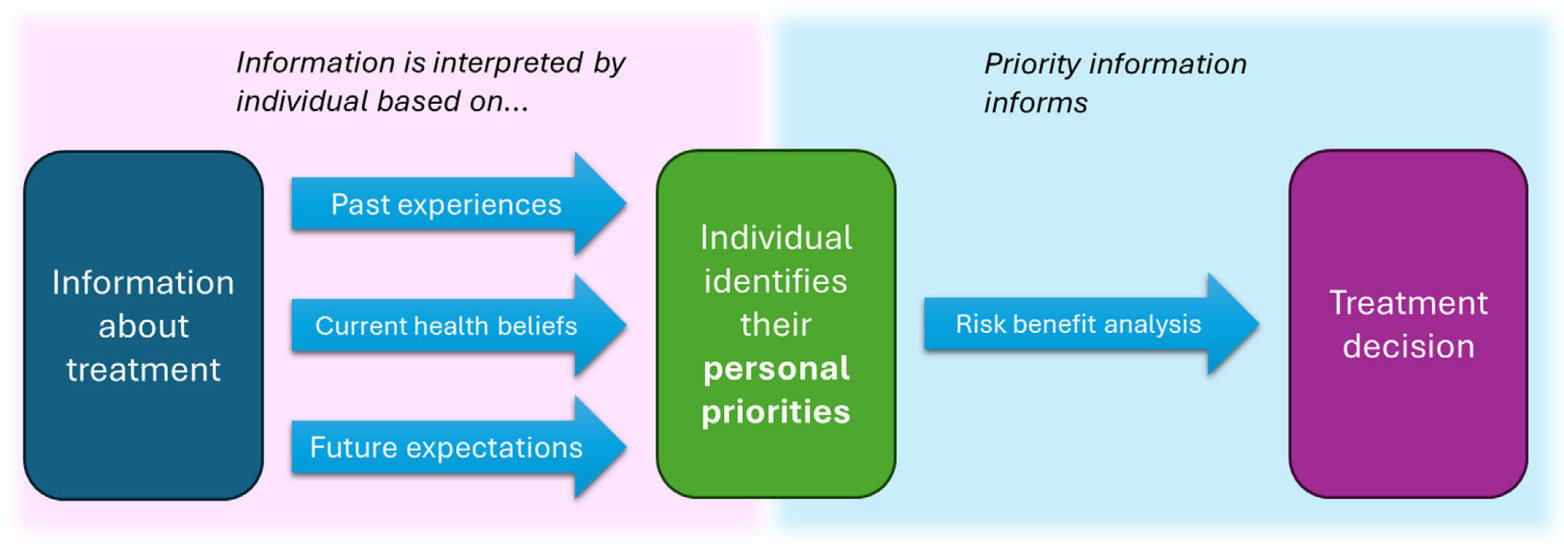
A diagram proposing a model to describe how individuals process information about treatments in order to make a treatment decision.

When describing the reasons for their choices and the factors men reported influenced their choice, a latent pattern was identified suggesting men's past experiences, present health beliefs, and future expectations influenced which treatment factors they prioritised. The high priority factors identified were then incorporated into each individual's risk–benefit analysis between different treatment options and ultimately impacted which treatment men chose (Fig. 2 and Table 3).

**Table 3:** Examples of participants reporting which factors influenced their treatment choice, demonstrating influence from past experiences, current health beliefs, or their expectations for the future.

Past experiences	‘The patch has got less chances of reducing bone density, and that's already my problem’. P8 ‘I’ve also had a little bit of experience of patches before for other medication, and I’ve have found they can be a bit uncomfortable and itchy’. NP4 ‘I already have an existing heart condition, so if there was something which offered some benefits from a cardiac side…’. NP3 ‘I’m used to already taking a B12 injection every 3 months and it's no imposition’. NP2
Current health beliefs	‘The constipation, the diarrhoea, I don’t think that would be a great issue’. P6 ‘There's no guarantee I would absorb the medication the same way as everybody else’. P7 ‘The heart issues I could see them being mitigated by a healthy lifestyle, I mean I’m 59 now, but I go to the gym regularly. I get out my bike, so I get plenty of exercise. So I kind of hope I’m already fighting the good fight against heart problems’. P4
Future expectations	‘The biggest fear I had was the side effects’. P4 ‘I rejected the injections straight away because I don’t want any more problems in my brain than I’ve already got’. P1 ‘I am a bit averse to jabs’. P13 ‘I wouldn’t want the patches though because they’re too obvious’. NP3

NP(participant number), from the group of men who never had prostate cancer; P(participant number), from the group who have or previously had prostate cancer.

### Theme 3: anticipated adverse effects of treatment (including side‐effects, treatment delivery and psychological impacts) influence which option is preferred

Many men reported the risk of side‐effects influenced their treatment decisions; however, different side‐effects were reported as important by different men. Generally, men seemed to perceive the side‐effects from injections to be greater than the side‐effects from the other treatments, even though this is unlikely to be the case for most side‐effects.

Most men identified the possible reduced risk of cardiovascular issues with tablets as an attractive feature of this approach, whilst fewer men reported interest in the improvement in metabolic parameters associated with the patches. This may reflect the asymptomatic nature of metabolic disturbance, even though metabolic changes may subsequently affect cardiovascular health. However, men in this study who already had hypertension and diabetes were more likely to identify improvement in metabolic endpoints as a reason for choosing patches. Furthermore, some men felt that difference in the cardiovascular risks and metabolic changes between treatments did not impact their choices, as these were factors that could be ameliorated by maintaining a healthy diet and exercise regimen.

Side‐effects related to testosterone suppression were also identified as important factors influencing choice. Most men reported that a more rapid return of testosterone to normal levels, and thus resolution of hormone side‐effects, would be a positive factor. Some men reported it would be important for them to avoid hot flushes, which can impact many aspects of daily life. Other men identified the risk of gynaecomastia as the deciding factor that would influence their treatment choice, describing aspects of body image and feelings of manliness being impacted by a treatment that increased breast tissue.

Additional psychological impacts of treatment included the concern that wearing a patch was a physical reminder of a cancer diagnosis and also visible to others, which could be stigmatising. Conversely some men reported that the visibility of the patch would reassure them that they are receiving their treatment. Similarly with tablets, some men felt that opening the package and taking a tablet might act as an unwelcome reminder of their cancer diagnosis. Finally, some men reported fear of injections as a reason for rejecting this as a preferred treatment option.

Other specific side‐effects were mentioned by particular individuals who identified aspects that were concerning to them, e.g., bone health in a participant with osteoporosis, or skin irritation from patches in a participant who had used nicotine patches.

### Theme 4: logistical issues (control, convenience, confidence, and cost to the health system) influence which option is preferred

Many participants reported logistical factors as major influences of treatment choices, with some men saying that these issues were more important to them than side‐effects. Logistical factors were further categorised into issues of control, convenience, confidence, and cost.

Participants preferred treatment options where they felt they would be in control of their treatment. One participant commented that applying a patch felt like they were actively participating in their treatment, and many reported being in control of their treatment was desirable.

Many participants reported difficulty accessing timely appointments from their GP surgery as a concern with the injections, reducing the feeling of control over their treatment. The necessity for attending their GP surgery every 12 weeks was also identified as inconvenient, taking up both time and money. On the other hand, self‐administered tablets or patches were perceived as a familiar, easy option that was convenient for many men. There was concern raised about the current patch regimen, with the need to apply multiple patches simultaneously seen as inconvenient, and men reported that patches would be more attractive if there was a single patch option.

Some participants reported concerns around specific situations that reduced their confidence in the effectiveness of treatments. Two men with chronic gastrointestinal issues reported they were not confident that they would absorb oral medications and therefore would not choose tablets. Some men reported concerns that the patches might not stick well, this was often mentioned in men who reported they went to the gym or swum regularly. The two men who chose injections as their first‐choice treatment both reported receiving a long‐acting treatment would give them confidence in their treatment and be reassuring.

Cost to the health care system was not discussed during the introductory presentation regarding the three treatments. This is not information that would be presented to an individual making a treatment choice in the UK, although this is likely to be different in other parts of the world. However, if the topic arose during the FGD, then it was mentioned that in the short‐term, it is likely tablets will be more expensive for the NHS in comparison to the other treatments. In response to this, many men reported that if they had been aware of this, they would not have chosen the tablets as their first option and would be happy to receive one of the other two forms of treatment.

### Theme 5: advice from trusted others supports men making treatment decisions

When participants were asked about the factors influencing their treatment decision, they did not explicitly report advice from others as a significant factor. In fact, when asked directly if advice from others influenced their choice, most men reported that it did not. However, particularly in discussions when past experiences of health care treatment decisions were being considered, many participants reflected on information and advice they had received as being helpful.

Some men reported they would prefer to be guided by the advice of a clinician with regards to this treatment decision. Men reported a preference for receiving advice from a specialist (urologist or oncologist) rather than a GP. Men reflected on previous experiences where they felt they had received good or poor advice from clinicians, and this often influenced the extent to which they wished clinicians to be involved in treatment decisions.

Some men reported it would be important for them to discuss the options available with their family, most frequently a discussion with their partner, although different weight was given to their partners opinion by different men.

Men who had prostate cancer treated previously, reported that resources and information from trusted websites including Macmillan, Prostate Cancer UK, and Cancer Research UK were helpful. One participant discussed how he used a helpline service where he was able to discuss his treatment options with a Prostate Cancer Nurse Specialist, which he found particularly helpful. Conversely, most men reported scepticism about the importance of celebrity stories, on‐line chat rooms, and social media, where it was felt information was not reliable.

## Discussion

This is the first study to explore how men would choose between these three types of ADT treatment. Importantly, the study demonstrated that men wished to have a choice of treatments available in this setting. Participants reported having choice and being involved with treatment making decisions would give them agency over their healthcare, and this could potentially reduce treatment regret. In addition, most men reported they would not choose standard subcutaneous LHRH agonist injections if they were counselled about these three ADT options and offered a choice between them.

Men reported their decisions were influenced by a wide range of factors, which were grouped into three categories: side‐effects, logistical aspects, and advice from trusted others. Different men prioritised different aspects of treatment as most important to them, resulting in a variety of choices. Therefore, if a choice of ADT becomes available, appropriate resources to support men make these decisions will be essential.

Furthermore, if all treatments were accessible then this would allow men who were struggling with any initial modality of ADT to switch to an alternative method. Having more choices in this regard could help some men persist with ADT who are otherwise experiencing intolerable side‐effects.

Most men reported that the rapid resolution of testosterone to normal levels seen with oral LHRH antagonists was attractive. None of the participants mentioned concerns around adherence with taking a daily medication, as opposed to an infrequent injection as a potential barrier. However, it is possible that consistently poor adherence to treatment combined with rapid recovery of testosterone could result in suboptimal castration and impact prostate cancer outcomes.

Sexual side‐effects were not discussed in detail in this study, on the premise that if castration is similar between treatments, sexual impacts are also likely to be similar. Furthermore, this sensitive topic may be difficult for participants to feel comfortable discuss in a FGD setting. However, an analysis of self‐reported quality of life data from participants in the PATCH trial suggested men treated with tE2 were less likely to report a ‘lack of sexual interest’ compared to men taking LHRH agonists (59% vs 74%) [14]. This may be an important possible benefit for some men. Further research exploring patients’ partners perspectives of these ADT options could also elicit additional informative data.

Although no comparative health economic assessment of these three treatments has been published to date, during FGDs it was discussed that the drug cost of oral LHRH antagonists would be higher than that of the other two treatment options discussed. However, since our FGDs, relugolix has become available in the UK at an annual cost of ~£1065, only moderately higher than that of injections (annual cost of Zoladex ~£940) and patches (annual cost of Progynova ~£552).^1^ With less disparate costs it is not clear whether this would still impact men's treatment decisions in a public health care system. Nonetheless, the use of a more expensive treatment has resource implications for health care systems, and where patients pay directly for their treatment, is likely to be a significant factor influencing treatment choice.

Our results, exploring the factors that influence treatment decisions, are consistent with other studies evaluating men's choices between different treatments in early prostate cancer. Discrete choice or standard gamble studies have found that men place different value on different aspects treatments [15, 16]. However, whilst there are a wide range of factors, with variability in how men prioritise these, the list of potentially important factors is finite and similar across multiple populations. A systematic review of international studies assessing factors and information that influence treatment decisions in early prostate cancer identified similar factors to this study. The review found men weigh treatment benefits vs side‐effects, with different men having different priorities for important benefits or side‐effects, whilst also considering the logistics of treatment (described as treatment processes in the review), confidence in, and ease of access to, their health care system, and decision assistance from others [17].

Results from qualitative studies should not necessarily be considered transferable to a wider population, and the findings could be different if the study was repeated in different countries or health care systems. Furthermore, a decision made in a hypothetical scenario discussion may not accurately reflect how these men would choose if confronted with the same decision in real life. However, a range of opinions was heard, exploring participants’ views of these treatments in some detail and giving insight into the types and breadths of concerns around these treatments. Focus group studies allow participants to share ideas, experiences, and views, stimulating discussion, but are reliant on participants being actively involved, responses may be influenced by social desirability, and discussion may silence single voices of dissent [18, 19]. Whilst most discussions elicited even contributions from participants, in one FGD in this study a single expert participant led much of the discussion, which may have influenced the responses of other participants.

Advertising via prostate cancer and health care research websites may have biased recruitment toward participants who are more engaged with the prostate cancer community, take an active interest in medical developments, and who are more involved with their own treatment and care [20]. During the discussions it was clear that a couple of men held pre‐existing strong views, including around ADT and patient choice, which may have influenced their decision to join the FGDs. However, all men were respectful of each other's opinions and differing views were expressed during the meetings.

Conducting on‐line FGDs facilitated recruitment of a geographically diverse population, although could potentially bias the group of respondents to a more affluent or technologically savvy group of participants [21, 22]. Previous comparisons of audio‐visual synchronous on‐line focus groups to face‐to‐face focus groups have found that although participants felt conversation flowed less freely in the on‐line format, they were comfortable to express their points of view, and data quality are similar regardless of approach [23, 24, 25]. During FGD sessions, conversation flowed well with all participants contributing meaningful opinions.

It is difficult to tease out the impact of possible recruitment and selection biases on the findings on this study. Research employing different methods, such as surveying wider representative groups of men, to further quantify the importance of factors identified from the study, could provide additional value.

The primary researcher (H.L.R.) was introduced as medical oncology doctor and clinical research fellow involved with two large clinical trials evaluating treatments for prostate cancer. However, it was not explicitly revealed that the prostate trials included studies investigating tE2 patches. It was felt that this would potentially bias discussions, drawing focus onto the tE2 option that may not have arisen naturally, or provoking a reluctance to criticise this treatment option. Participants in FGDs respond differently depending on who leads the discussion [26], consequently the characteristics of the primary researcher (including being White, female, a medical professional, and younger than participants) may have affected the information participants felt comfortable sharing. Therefore, all FGDs were co‐facilitated by relatable facilitators with the PPI representatives. This created a comfortable environment, with feedback after each session from the PPI facilitators reporting active engagement from all participants.

Themes generated during analysis may be influenced by the background of the primary researcher, reflecting the medical perspectives of treatment decision‐making and from experiences working within a trial comparing tE2 to LHRH analogues (although the results of the trial were not known at the time of the FGDs). Coding clinics including researchers not involved with either the PATCH or STAMPEDE trials and non‐clinical researchers provided additional insight and perspectives to the data. In addition, the PPI co‐facilitators were asked to review the themes and felt these were appropriate.

In conclusion, this focus group study found that most men who participated felt having choice of ADT was important and that it could lead to tangible benefits for patients. The majority of men reported they would prefer to receive either tablet or patch options for ADT, citing a wide range of factors including the different side‐effect profiles, logistical aspects of treatment, and advice from trusted others as influencing these choices. Participants reported that their decisions may differ depending on the treatment setting and duration of treatment proposed, highlighting the importance of providing clear, tailored information addressing the men's personal priorities at the time of the decision, to support decision making. Those developing guidelines for management of men with advanced prostate cancer should consider these points of view expressed by participants within this focus group study when updating ADT recommendations.

## Disclosure of Interests

Hannah L. Rush: grants or contracts from any entity: Medical Research Council (MRC) Clinical Trials Unit at University College London (UCL). Sophie Merrick: grants or contracts from any entity: MRC Clinical Trials Unit at UCL. Nicholas D. James: grants or contracts from any entity: funding from Cancer Research UK and Prostate Cancer UK for trial conduct and translational sub studies (institutional). Duncan C. Gilbert: grants or contracts from any entity: MRC Clinical Trials Unit at UCL. Annabelle South: grants or contracts from any entity: MRC Clinical Trials Unit at UCL. Ruth E. Langley: grants or contracts from any entity: MRC Clinical Trials Unit at UCL. No other authors report relevant conflict of interests.

## Funding

This study received funding for participant reimbursement from the PATCH trial, which has grants from Cancer Research UK (grant number C17093/A12443; trial CRUK/06/001) and the MRC (MC_UU_00004/02). The funding sources had no role in the study design, conduct, analysis, or interpretation of the data; preparation, review, or approval of the manuscript; nor decision to submit the manuscript for publication.

## Supporting information


**Appendix S1.** Focus group discussion agenda.
**Appendix S2.** Slides used in initial presentation during focus group discussions.
**Appendix S3.** Standards for Reporting Qualitative Research (SRQR) checklist.

## Footnotes

^1^
Costs based on those in the on‐line British National Formulary (BNF) accessed on the 20 June 2025.
